# Rapid Biodegradation Assessment of Biomass Plastics Using Closed Recirculating Aquaculture Systems: A Novel Approach for Environmental Sustainability

**DOI:** 10.1007/s10126-025-10436-x

**Published:** 2025-02-28

**Authors:** Wilasinee Kotcharoen, Naoki Wada, Makiko Kakikawa, Noboru Takiguchi, Yoshinori Takahashi, Kenji Takahashi, Yutaka Takeuchi

**Affiliations:** 1 Faculty of Biological Science and Technology, Kanazawa University, Kanazawa, Ishikawa 920-1192 Japan; 2 Faculty of Frontier Engineering, Kanazawa University, Kanazawa, Ishikawa 920-1192 Japan; 3 Central Research Institute, Maruha Nichiro Corporation, 16-2, Wadai, Tsukuba-City, Ibaraki 300-4295 Japan

**Keywords:** Biomass plastic, Early decomposer, Biodegradability, PBSA, Recirculating aquaculture system

## Abstract

The increasing plastic production causes serious problems in the marine environment, and the main source of plastic waste comes from the fishing and aquaculture industries. Although there have been various efforts to develop aquaculture equipment with marine biodegradable plastics, an urgent need is to develop an assay to evaluate their biodegradation in aquaculture environments. This study focused on evaluating the biodegradation of biomass plastic in recirculating aquaculture systems (RAS) that mimic freshwater, brackish water, and saltwater aquacultures. The methods used to assess biomass plastic biodegradability included changes in physical properties, weight loss, biochemical oxygen demand, and microbial community investigation using poly(butylene succinate-co-adipate) (PBSA) as a model. Scanning electron microscopy studies indicated the erosion on the biomass plastic surface from 1 to 2 days in the RAS tank (salinity, 0–0.5%) harboring Nile tilapia (*Oreochromis niloticus*). 4′,6-Diamidino-2-phenylindole fluorescence microscopy confirmed the presence of the microorganisms on the PBSA surface. The microorganisms in RAS tanks degraded 11.6% of 1 g/L PBSA in 7 days, demonstrating their biodegradation potential. 16S rRNA gene sequencing showed that *Pseudomonas* plays a major role as an early decomposer in the biodegradation process within 24 h. A multifaceted analytical method that provides sufficient evidence was developed to show that the erosion on the PBSA surface in RAS tanks results from biodegradation. The ability of RAS to control various aquatic environments (pH, salinity, temperature, and bacterial density) makes it suitable for testing the marine biodegradability of biomass plastics for use in aquaculture and fishery industries.

## Introduction

About 400 million tons of plastics are produced worldwide and end up in the ocean at least 8 million tons annually (UN 2023; Omura et al. 2024). The increasing plastic production causes serious environmental problems (Huang et al. 2021) and impacts marine ecosystems, food and human health, tourism, and climate change. Plastic waste is the most abundant type (80%) of waste in the ocean, which is found in surface waters and deep-sea sediments (IUCN 2021). At open-net-cage and closed aquaculture sites, plastics are washed out to the sea due to damage caused by rapid tides and typhoons, and ~ 50% of plastic debris washed ashore is fishery garbage. Over time, some plastic debris descends to the deep sea due to increased weight caused by biofouling or sand grain accumulation on the plastic surface, making it denser than seawater (Omura et al. 2024). Some plastics break down into small particles, so-called microplastics (< 5 mm) or nanoplastics (< 100 nm), which are easy for marine wildlife (i.e., seabirds, whales, fish, and turtles) to ingest accidentally and die of starvation (IUCN 2021; Walker 2021; Phothakwanpracha et al. 2021; Soe et al. 2024). In addition, microplastics have been found in tap water, beer, and salt, which affects food quality and human health (IUCN 2021). In aquaculture sites, microplastics accumulate in aquatic animals (i.e., shells, fish, and shrimp). Most plastics are produced from petroleum-based materials and are nonbiodegradable (Huang et al. 2021), such as polyesters or polyolefins, which accumulate for long periods in the environment (Slezak et al. 2023). Thus, there is a need to change the plastic material from petroleum-based and nonbiodegradable to biodegradable.

In recent years, various biodegradable polymers, also known as biomass plastics or bioplastics, such as polycaprolactone (PCL), poly(L-lactic acid) (PLA), poly(butylene succinate) (PBS), poly(butylene adipate) (PBA), and poly(butylene succinate-co-adipate) (PBSA), have been widely studied and implemented to solve severe plastic pollution problems (Lee and Kim 2010). Bioplastics can undergo mineralization into carbon dioxide (CO_2_), methane, water, inorganic compounds, or biomass through enzymatic activity from specific microorganisms such as bacteria and fungi under suitable environmental conditions (Tamnou et al. 2021). These microorganisms secrete exoenzymes that break down chemical bonds such as ester, glycosidic, and peptide bonds, facilitating biodegradation (Lee and Kim 2010). Efforts to incorporate marine biodegradable plastics into fishing and aquaculture have gained attention in recent studies as a potential solution for reducing plastic pollution from lost or discarded products in the ocean. Examples include biodegradable feeding trays (Carfì Pavia et al. 2023) and fish cages, as well as polybutylene succinate-co-adipate-co-terephthalate (PBSAT) snoods tested in the Barents Sea longline fishery to replace nylon. Trials showed no significant short-term differences in capture efficiency or snood loss rates between PBSAT and nylon (Cerbule et al. 2022, 2023). The biodegradability of these polymers depends on their composition, chemical structure, physical properties (such as mechanical strength and crystallinity), and susceptibility to microbial degradation (Lee and Kim 2010). Understanding these factors is crucial for designing bioplastics suitable for marine applications while ensuring environmental sustainability. However, no method has been developed to assess marine biodegradability in aquaculture environments. Theoretically, microorganisms are more abundant in aquaculture environments where fish are kept at high densities, and biodegradation is expected to occur more rapidly than in nature and/or deep water. Because biodegradation is directly related to the degradation of aquaculture equipment, it is important to accurately assess the degree of biodegradation in the aquaculture environment. From a different perspective, the quick test could be developed using the aquaculture environment for biodegradation testing.

In this study, PBSA was chosen as an aliphatic polyester, one of the most alternative biodegradable plastics investigated and developed on an industrial scale (Huang et al. 2021) because its mechanical properties are stronger and the production cost is lower than other biodegradable polymers (Hayase et al. 2004). Moreover, due to its lower crystallinity, the PBSA copolymer is characterized by a higher degradability by enzymatic processes than other materials (Puchalski et al. 2018). PBSA is manufactured at a semicommercial scale through transesterification and polycondensation involving 1,4-butanediol, succinic acid, and adipic acid. The PBSA structure consists of randomly copolymerized units of butylene succinate and butylene adipate in its main chain (Lee and Kim 2010). Bacteria that can degrade PBSA are found extensively in soils, compost, and activated sludge (Hayase et al. 2004). Several enzymes derived from fungi and bacteria, such as polyester depolymerases, esterases, cutinase-like enzymes, and lipases, are involved in aliphatic polyester degradation. Among these enzymes, lipases can catalyze the hydrolysis of ester linkages in different polyesters (Urbanek et al. 2020; Huang et al. 2021). Microorganisms, such as *Burkholderia cepacia* and *Pseudomonas aeruginosa*, secrete lipase, which is responsible for the hydrolysis of aliphatic polyesters (Lee and Kim 2010). Hayase et al. (2004) reported that *Bacillus pumilus* can degrade PBSA at 100% degree of degradation, higher than that of PBS (90.2%), PBS/PCL blend (50.8%), and PLA (0.9%), at 30 °C. Recently, Kimura et al. (2023) discovered that *Vibrios* within the Gazogenes clade carry genes associated with a PBS-degrading enzyme. Moreover, *Vibrionaceae* and *Pseudoalteromonadaceae* exhibited substantial enrichment on films comprising PBS and PBSA blastospheres. The abovementioned biodegradation research was conducted in compost, soil, municipal solid waste, natural seawater, freshwater, and digestion (Emadian et al. 2017; Slezak et al. 2023). To the authors’ knowledge, the assessment of biomass plastic biodegradation using a RAS has not been established.

As a contemporary intensive aquaculture method, RAS operates at a high density and relies on recycling aquaculture water. This approach aims to achieve the efficient, environmentally friendly, and sustainable comprehensive utilization of natural resources (Li et al. 2023). The advantage of RAS is its ability to recycle aquaculture water by utilizing water treatment units for filtration and purification. The environmental conditions in RAS can be tightly controlled (Brauner and Richards 2020; Li et al. 2023). The nitrogen compound concentration in RAS is kept at a low concentration for fish survival; however, it is higher than natural seawater or freshwater. Another advantage of using RAS for the biodegradation test is that biomass plastic can provide the carbon source for bacteria surrounding biomass plastics as biofilm in the biodegradation process. Indeed, long-term feeding study in fish in closed RAS revealed that the total organic carbon (TOC) was lower than 80 mg/L and TOC/total nitrogen (TN) ratio are commonly lower than ~ 0.5, resulting in limitation of microorganism’s activities (Kotcharoen et al. 2021; 2023).

This study aimed to evaluate the microbial decomposition of biomass plastic in different 18 RAS fish reared, including Nile tilapia (*Oreochromis niloticus*), common carp (*Cyprinus carpio*), Honmoroko (*Gnathopogon caerulescens*), Indian shortfin eel (*Anguilla bicolor pacifica*), longtooth grouper (*Epinephelus bruneus*), grass puffer (*Takifugu niphobles*), and sea urchin (*Anthocidaris crassispina*). The test was conducted at a water salinity of 0–36 ppt and water temperature of 25–29 °C. The morphological changes in the biomass plastic surface were evaluated by scanning electron microscopy (SEM) and 4′,6-diamidino-2-phenylindole (DAPI) staining. The microbial communities on the PBSA surface were investigated to understand the performance of PBSA degradation in the RAS environment.

## Materials and Methods

### RAS Tank

**Fig. 1:**
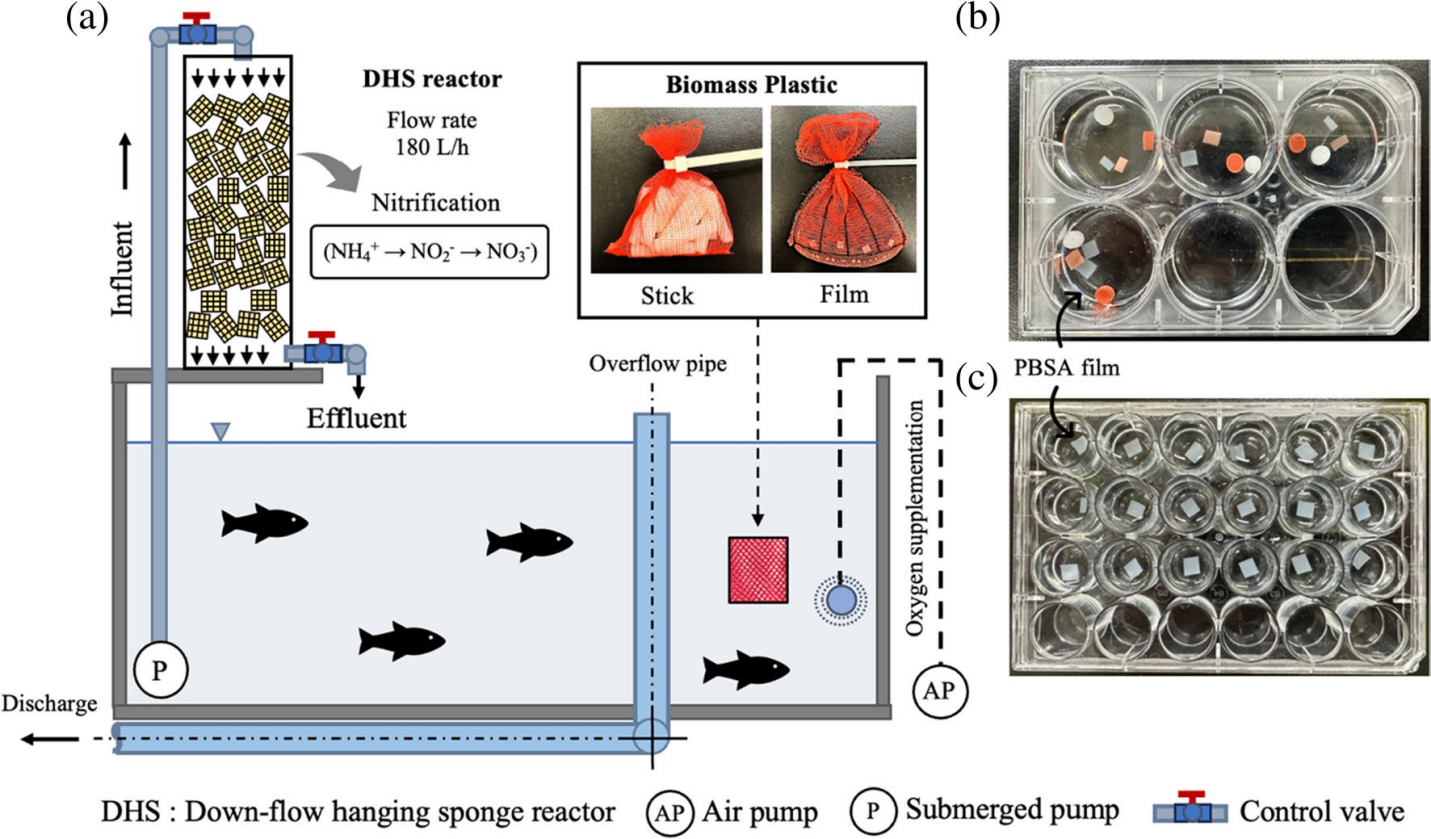
Flow diagram of the biomass plastic biodegradation in (**a**) a closed RAS, (**b**) a 6-well plate test, and (**c**) a 24-well plate test

Figure 1a shows a schematic of a laboratory-scale RAS under controlled temperature and 0.5% salinity. Twenty Nile tilapia (weight, 75 ± 4.5 g/fish) were reared in a 120-L closed RAS tank. Fish density was ~ 12.5 kg fish/m^3^. A downflow hanging sponge (DHS) reactor (diameter, 125 mm; height, 500 mm) was installed to remove ammonia nitrogen (NH_4_^+^-N) from the closed RAS (Kotcharoen et al. 2021). Polyurethane sponge (33 × 33 × 33 mm) media were randomly filled into the DHS reactor, and the total sponge volume was 2.26 L. The water pump flow rate was 180 L/h. The water quality in the RAS tank was maintained at optimum conditions for fish growth. The pH, dissolved oxygen, and water temperature in the RAS tank were 7.8 ± 0.3, 7.8 ± 0.6 mg/L, and 25 °C ± 1 °C, respectively. The nitrogen content in the RAS tank was maintained at an optimum range for fish growth; NH_4_^+^-N, NO_3_^−^-N, and NO_2_^−^-N were 0.2 ± 0.1, 40 ± 14, and 0.6 ± 0.6 mg/L, respectively, during the study.

### PBSA Sample for the Biodegradation Analysis

PBSA was supplied in a stick shape with an average diameter of 5 and 30 mm (Mitsubishi Chemical Co., Ltd.) for SEM analysis (“SEM” section), DAPI staining (“Fluorescence Microscopy” section), and biochemical oxygen demand (BOD) test (“BOD Measuring System” section). PBSA films were prepared at 5 mm width and 5 mm length for microbial community observations, plate tests, and weight loss. PBSA films were formed by a heat press machine (100 °C, 5 min) with 0.12 mm thickness. In this study, the PBSA sticks and films were added to the RAS tank (Fig. 1a).

### Surface Observation of PBSA in Tilapia RAS

The erosion on PBSA sticks and films was studied in the closed RAS tank (Fig. 1a). The samples were collected from the RAS tank every 24 h to observe the changes on the PBSA surface with SEM. To identify the microorganisms responsible for the erosion of PBSA samples, three sets of RAS water (WP1, WP2, and WP3) were prepared in six-well plates (Fig. 1b). WP1 was sterilizing water from the RAS tank. RAS water was sterilized at 121 °C, 2 atm, for 20 min in a high-pressure steam sterilizer (BS-325 TOMY Co., Ltd.) to inactivate bacteria, plankton, and other biological materials in the water sample. WP2 was RAS water containing 1% penicillin–streptomycin solution (Fujifilm Wako Pure Chemical Corp.). WP3 was water from the RAS tank. PBSA sticks were immersed in WP1 to WP3. The samples were collected every 24 h to observe the surface changes on the PBSA surface by SEM.

### SEM

Macroscopic observations of PBSA stick and film surfaces were performed using SEM (Hitachi TM4000Plus Miniscope, Japan). The PBSA film and stick samples were prepared by gently washing them with pure water and fixing them with Tissue-Tek U-fix (Sakura Finetech, Inc., Torrance, CA, USA) for 5 min. The samples were washed 3 times in pure water, dried at room temperature for ~ 5 min, and imaged using SEM.

### Fluorescence Microscopy

Microorganisms on the PBSA surface were observed using DAPI staining in combination with fluorescence microscopy (Olympus IX3-LHLEDC, Japan). Fluorescence microscopy images were obtained using FluoView FV3000 FV31S-SW Viewer software. PBSA films were collected from the closed RAS tank after degradation in the RAS tank for 48 h. The PBSA films were gently washed 1–3 times in pure water as needed. The staining solution was prepared by adding 1 µL DAPI stock solution in 1 mL rapid fixative (U-fix), protected from light. The stain solution was added to cover the PBSA film and incubated for 5 to 10 min. The film was washed 3 times in pure water to remove the stain solution and imaged.

### Optical Microscopy

The physical changes in the structure of PBSA films were photographed using an optical microscope (Olympus BX53F2, Japan). The films were collected from the RAS tank after 48 h to observe the changes in PBSA films. The PBSA film was placed on the slide, a small drop of RAS water was added to the film, the coverslip was put immediately after adding RAS water, and the film was imaged.

### BOD Measuring System

PBSA biodegradation was assessed by determining the BOD concentration to monitor microbial activity in the water according to the Organization for Economic Co-operation and Development (OECD) guideline 301F (Stasinakis et al. 2008; Takekoshi et al. 2022). BOD concentration was determined using a respirometry activity sensor system (6 BIO.P, VELP® Scientifica, Italy). Within the systems, microorganisms within a test bottle convert oxygen to CO_2_ as they biodegrade a test chemical. As a CO_2_ trap within the bottle absorbs the CO_2_, the system detects a reduction in the air pressure inside the bottle (Takekoshi et al. 2022). A respirometry activity sensor system (VELP®) is a cap-shaped pressure meter affixed to a test bottle. It assesses BOD without generating oxygen; instead, it detects the extent to which the air pressure in the bottle decreases (Reuschenbach et al. 2003). After a test bottle is sealed with a VELP® cap, the bottle is stirred and incubated in a temperature-controlled at 25 °C ± 1 °C (Takekoshi et al. 2022). The test RAS water volume was fixed at 100 mL, and the capacity of the light-resistant glass bottle was 500 mL. Two sets of PBSA sticks (1.0 and 10.0 g/L) were separately placed as the sole carbon source in the test bottles. The bottles without PBSA were set as blank samples in this study. BOD was performed in three test trials of 7, 14, and 21 days each. BOD data were recorded every 6 h throughout the test. The CO_2_ gets absorbed by absorbents (strong alkali positioned) in the neck of the bottle. After stopping the test, the absorbents were photographed immediately under a stereomicroscope (dissecting microscope) (Leica 10450028, Germany). Biodegradation can be calculated using Eq. (1), and the theoretical oxygen demand (ThOD) without nitrification can be calculated using Eq. (2) (OECD 1992):

1 $$Biodegradation\left(\%\right)=\frac{BOD_{sample}-BOD_{blank}}{ThOD}\times100$$

2 $$ThOD=\frac{16\left(2c+{\displaystyle\frac12}\left(h-cl-3n\right)+3s+{\displaystyle\frac5{2p}}+{\displaystyle\frac12}na-o\right){\displaystyle\frac{mg}{mg}}}{MW}$$

where BOD_sample_ is the BOD of RAS water with PBSA (mg/L); BOD_blank_ is the BOD of the blank control, RAS water without PBSA (mg/L); ThOD is the theoretical oxygen demand required (mg/L), and the ThOD used in this study was 1.72 (mg/mg); and MW is the molecular weight of the compound C_c_H_h_Cl_cl_N_n_Na_na_O_o_P_p_S_s_, and the ratio of succinate to adipate was 4:1.

### Weight Loss of PBSA Samples

Weight loss biodegradation is the most commonly analyzed factor for assessing material degradation (Puchalski et al. 2018). PBSA films were collected from the RAS tank every 24 h for 6 days, washed with pure water, wiped to remove excess water, and dried overnight to a constant weight at room temperature. The weight of the PBSA samples was measured by an analytical balance HR-251A (A&D Co., Ltd., Japan). The weight loss of the sample was the average of three sets of samples, which can be calculated using Eq. (3).

3 $$Weightloss\left(\%\right)=\frac{W_0-W_t}{W_0}\times100$$

where *W*_0_ is the initial weight of the sample before degradation in the RAS tank (mg), and *W*_t_ is the dry weight of the sample after degradation in the RAS tank at time *t* (mg).

### Microbial Community Analysis Using the 16S rRNA Gene

The PBSA samples were collected from the closed RAS tank at 24 h to identify bacteria responsible for most of the decomposers on the PBSA surface and on day 35 of biodegradation in the RAS tank. The sample was stored at 80 °C until DNA extraction. DNA was extracted using the Puregene® DNA tissue Kit (Qiagen) according to the manufacturer’s instructions. The extracted DNA concentrations were measured using a NanoDrop LITE spectrophotometer (Thermo Fisher Scientific, USA). Polymerase chain reaction (PCR) amplification of 16S rRNA genes was performed using the universal forward primer Univ515F (5ʹ-GTG CCA GCM GCC GCG GTA A-3ʹ) and reverse primer Univ806R (5ʹ-GGA CTA CHV GGG TWT CTA AT-3ʹ) (Caporaso et al. 2012). The PCR mixture was performed in a total volume of 20 µL consisting of 10 µL KOD One™ PCR Master Mix-Blue, 7 µL nuclease-free water, 1 µL forward primer, 1 µL reverse primer, and 1 µL DNA templates. PCR amplification was performed in a BIOER LifeECO™ Thermal Cycler PCR, with the following conditions: Stage I consisting of one cycle of initial complete denaturation (94 °C for 1 min) and Stage II consisting of 30 cycles of denaturation (94 °C for 15 s), annealing (55 °C for 15 s), and elongation (68 °C for 15 s). After 30 cycles, the temperature was maintained at 4 °C. The PCR sample was analyzed using 16S rRNA gene sequencing using MiSeq (Illumina, Inc., USA). Raw sequencing data were analyzed using QIIME 2 (Bolyen et al. 2019). Operational taxonomic units were classified with ≥ 97% similarity using Silva database version 138 (Glöckner et al. 2017). 16S rRNA sequences were identified using a web-based BLAST search of the National Center for Biotechnology Information database (https://blast.ncbi.nlm.nih.gov/Blast.cgi).

### Multiwell Plate Test for Quick Screening of RAS Water with High Biodegradation Activity

In this study, quick tests for PBSA biodegradation under various aquaculture conditions were performed in 24-well plates (Fig. 1b). RAS water was collected from 18 different RAS tanks consisting of Nile tilapia (8 tanks), Honmoroko (1 tank), black carp (1 tank), colored carp (2 tanks), eel (1 tank), longtooth grouper (1 tank), sea urchin (1 tank), and grass puffer (3 tanks). PBSA samples were collected, and erosion holes were investigated by optical microscopy after biodegradation in 24-well plates for 48 h.

## Results

### Morphological and Weight Changes in PBSA Immersed in RAS

**Fig. 2:**
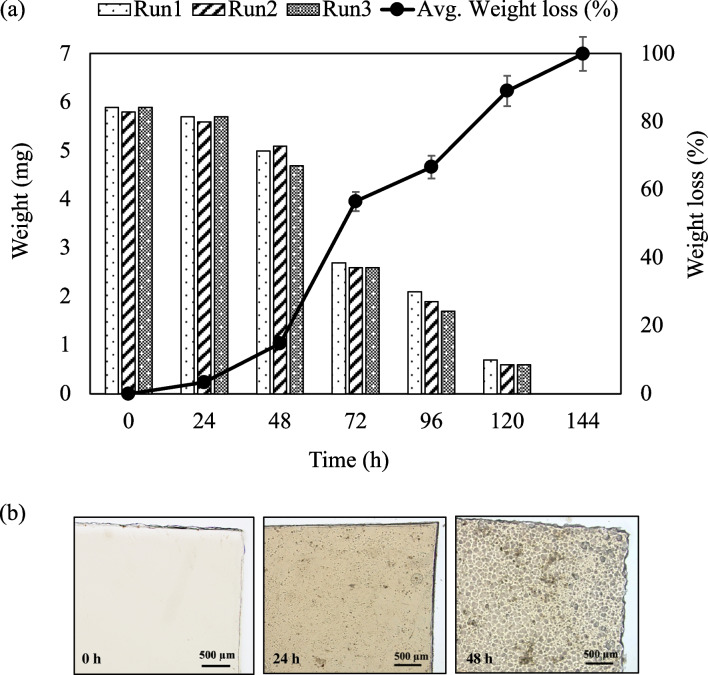
(**a**) Weight loss plots of PBSA films immersed in the 120-L closed aquaculture system over time and (**b**) optical microscopy image of PBSA films at 0, 24, and 48 h

Figure 2 shows the weight loss of PBSA films during biodegradation in the RAS tank. Weight loss was measured after 24, 48, 72, 96, 120, and 144 h in the RAS tank. The average weight of the PBSA film was 6.1 ± 0.3 mg. After 24 h, small holes were observed on the PBSA film surface, and weight loss was ~ 3.4 ± 0.1%. Weight loss increased to 14.8% ± 1.9%, 56.6% ± 0.7%, 66.7% ± 2.6%, and 89.1% ± 0.9% after biodegradation in the RAS tank for 48, 72, 96, and 120 h, respectively. Results showed a weight loss of 100% after 144 h. The average weight loss of the PBSA film in the RAS tank was 0.8 ± 0.4 mg/day. The optical microscopy image showed physical deformation on the PBSA film at 0, 24, and 48 h (Fig. 2b). PBSA films degraded rapidly, prompting the use of PBSA sticks for long-term studies, including SEM observations and BOD tests.

**Fig. 3:**
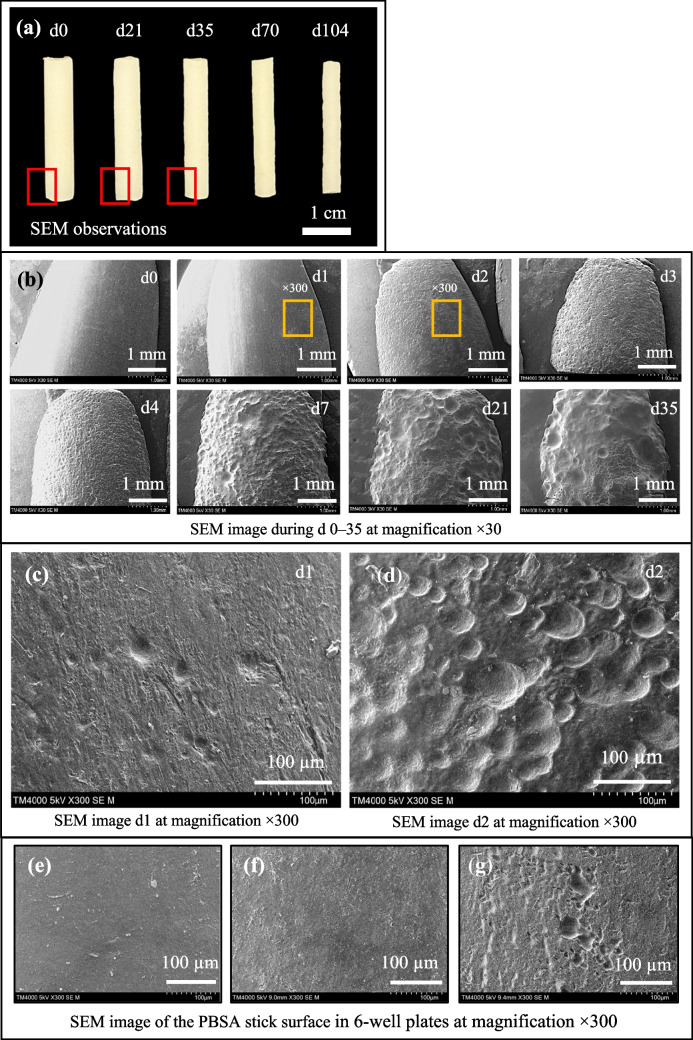
(**a**) Photographs of PBSA sticks before and after biodegradation in RAS. (**b**) SEM image of the PBSA stick surface at × 30 magnification on days 0 to 35 and × 300 magnification on (**c**) day 1 and (**d**) day 2 and SEM image of the PBSA stick surface at × 300 magnification after immersion in 6-well plates. (**e**) Autoclave RAS water (WP1), (**f**) antibiotic control (WP2), and (**g**) RAS water (WP3) for 48 h

Figure 3 shows the surface changes of PBSA sticks during the study. Figure 3a shows the physical changes of PBSA sticks on days 0, 21, 35, 70, and 104. Based on the photographs, the erosion on the biomass plastic surface caused physical deformation, as shown by the roughness and decreased thickness of PBSA sticks. Figure 3b shows the SEM observations of the PBSA stick surface before and after degradation in the RAS tank from days 0 to 35. SEM images showed that the surface before degradation (d0) was smooth, with erosion noticeable by small holes on the PBSA stick surface at 48 h. However, when observing the SEM image at higher magnification (× 300), as shown in Fig. 3c, small holes were visible on the PBSA surface at 24 h, which gradually enlarged as degradation progressed from days 1 to 35.

**Fig. 4:**
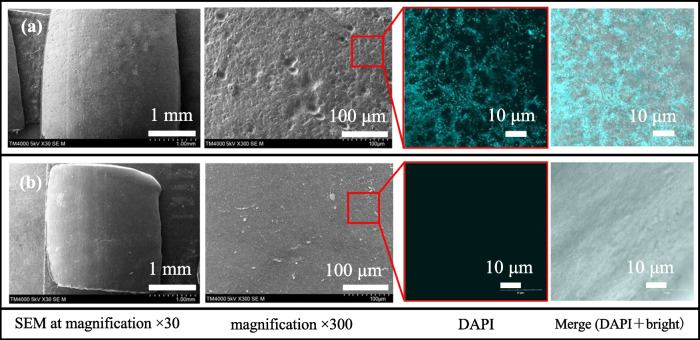
Microscopic observation of the PBSA stick surface using SEM and DAPI staining: (**a**) fresh RAS water and (**b**) autoclave RAS water after immersion in a 6-well plate for 48 h

Plastic polymers can undergo degradation to different extents through physical and biological processes (Tamnou et al. 2021). To understand the reason for the erosion on the biomass plastic surface caused by bacterial activities, the erosion of the 48 h biodegradation PBSA surface in the six-well plate, including the autoclave RAS water (WP1), antibiotic (WP2), and RAS water (WP3), was also observed. SEM images showed that the PBSA surface on WP1 and WP2 demonstrated no changes due to erosion (Fig. 3e and f), whereas the erosion in WP3 had roughness and many holes on the surface (Fig. 3g). In addition, bacteria in the bottom of the hole on the PBSA surface in WP3 were detected by DAPI staining (Fig. 4a), whereas bacteria in WP1 were not observed (Fig. 4b), indicating that bacteria are proven to cause erosion on the PBSA surface. Based on these results, degradation resulted from microbial processes rather than physical deformation.

### Real-Time BOD Test Using the Respirometry Activity Sensor System

**Fig. 5:**
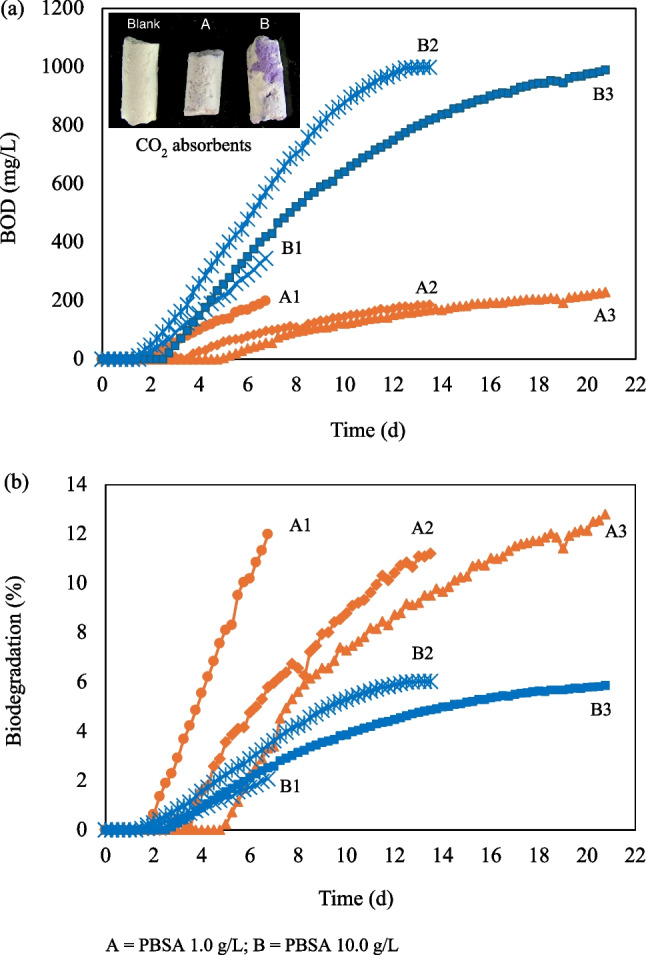
(**a**) BOD curves and (**b**) PBSA biodegradability using RAS water

PBSA biodegradability was determined by evaluating the BOD concentration in a respirometry activity sensor system at a controlled temperature of 25 °C ± 1 °C using PBSA sticks as the sole carbon source in RAS water. Figure 5 shows the BOD concentration recorded by a respirometry activity sensor system and the degree of biodegradation in trial 1 (A1 and B1; 7 days), trial 2 (A2 and B2; 14 days), and trial (A3 and B3; 21 days). The conditions of RAS water, pH, and DO values are shown in Table 1. The test RAS water without PBSA (blank) showed no sign of CO_2_ production (BOD, 0 mg/L) throughout the experiment (data not shown). BOD in RAS water with 10 g/L (B1–B3) started to increase on days 1.75, 1.50, and 2.75 in trials 1–3, respectively, earlier than 1 g/L (A1–A3), and BOD started on days 1.75, 2.75, and 5.0 in trials 1–3, respectively (Fig. 5a). In trial 1, where the experiment was stopped on day 7, BOD of A1 and B1 was 199 and 345 mg/L, corresponding to 11.6% and 2.0% biodegradation, respectively (Fig. 5a and b). In the 1 g/L group (A2–A3), the experiment with a longer incubation time did not increase BOD over 200 mg/L and degree of biodegradation. In the 10 g/L group (B2–B3), a longer incubation time increased the degree of biodegradation to 5.8% (B2) and 5.7% (B3), and BOD became stable at ~ 999 mg/L, reaching the maximum BOD concentration in this condition (for a sample volume of 100 mL). As the CO_2_ trap within the bottle absorbs the CO_2_, the absorbents turn from white to purple after the experiment, confirming their degradation to CO_2_ in the BOD bottle (Fig. 5a).

**Table 1:** Conditions of RAS water, pH, and DO before and after biodegradation in the real-time BOD test

Trial	pH (average ± SD)			DO (average ± SD)		
	Before	After		Before	After	
		1 g/L PBSA	10 g/L PBSA		1 g/L PBSA	10 g/L PBSA
1	7.6	7.9 ± 0.01	7.9 ± 0.1	8.69	7.13 ± 0.04	3.44 ± 0.20
2	6.9	7.8 ± 0.1	8.3 ± 0.1	8.83	7.37 ± 0.21	3.30 ± 0.25
3	7.6	8.8 ± 0.2	8.5 ± 0.1	8.46	6.33 ± 0.06	0.73 ± 0.04

Trial 1, 7 days; trial 2, 14 days; trial 3, 21 days; *n* = 2

### Microbial Community Structure on the PBSA Surface

**Fig. 6:**
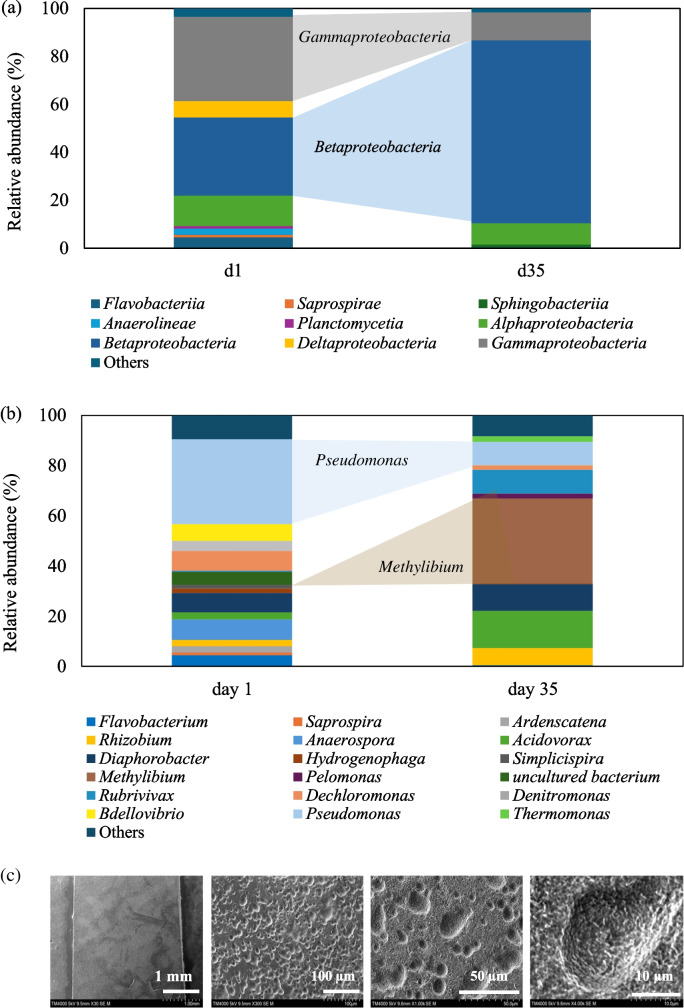
Microbial community: (**a**) class level and (**b**) genus level on the PBSA surface at 24 h in the RAS tank and (**c**) PBSA film surface observed by SEM at × 30, × 300, × 1000, and × 4000 magnification

DNA was extracted from the films when the holes were first identified on day 1 and compared to the microbial community structure when severe erosion was observed on day 35. The microbial community structure on the PBSA surface is shown in Fig. 6. 16S rRNA gene analysis showed that *Proteobacteria* was the most abundant phylum detected on the PBSA surface. The relative abundance of *Proteobacteria* was 87% and 97% on days 1 and 35, respectively (data not shown). Figure 6a shows a clear difference in microbial communities by class between days 1 and 35. Classes *Gammaproteobacteria* (35%) and *Betaproteobacteria* (33%), which belong to the phylum *Proteobacteria*, were the most dominant on the PBSA surface on day 1. The relative abundance of the class *Gammaproteobacteria* decreased to 12%, whereas *Betaproteobacteria* increased to 76%, the most dominant class on day 35. Figure 6b shows the relative abundance by genus level on days 1 and 35. Results showed a high relative abundance of *Pseudomonas* (33.75%) on day 1. The relative abundance of *Pseudomonas pseudoalcaligenes* and *Pseudomonas stutzeri* was 32.51% and 1.24%, respectively. On day 35, *Methylibium* (33.74%) was the most dominant genus, whereas *Pseudomonas* had 9.26%.

### Quick Biodegradation Test in a Multiwell Plate Using Water from Various RAS Tanks

**Fig. 7:**
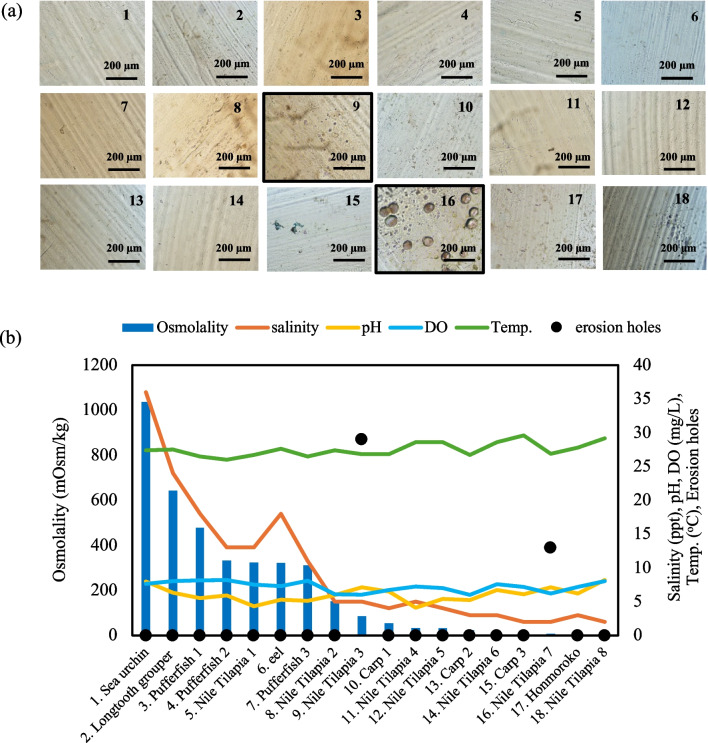
Erosion test in a multiwell plate using water from different RAS tanks: (**a**) PBSA surface at 48 h by optical microscopy and (**b**) the conditions in RAS tanks: (1) sea urchin (*Echinoidea*); (2) longtooth grouper (*E. bruneus*); (3) pufferfish (*Tetraodontidae*), tank 1; (4) pufferfish, tank 2; (5) Nile tilapia, (*O. niloticus*), tank 1; (6) eel (*Anguilliformes*); (7) pufferfish, tank 3; (8) Nile tilapia, tank 2; (9) Nile tilapia, tank 3; (10) carp (*C. carpio*), tank 1; (11) Nile tilapia, tank 4; (12) Nile tilapia, tank 5; (13) carp, tank 2; (14) Nile tilapia, tank 6; (15) carp, tank 3; (16) Nile tilapia, tank 7; (17) Honmoroko (*G. caerulescens*); and (18) Nile tilapia, tank 8

Figure 7 shows the changes on the surface of PBSA sticks after biodegradation in 18 conditions of RAS water for 48 h. The hole on the surface showed signs of biodegradation in tilapia tank 3 (29 holes) and tank 7 (13 holes) (Fig. 7a), and holes on PBSA in any other tanks were not observed. Figure 7b shows the different RAS rearing and environmental conditions in the tanks. However, the conditions in Nile tilapia tanks 3 and 7 exhibited similar values. The pH values in tanks 3 and 7 were 7.1, the average water temperature was 26.9 °C ± 0.1 °C, and DO was 6.1 ± 0.1 mg/L. The osmolality of tilapia tanks 3 and 7 were 8 and 84 mOsm/kg, respectively. Salinity in tilapia tanks 3 and 7 were ~ 0.2% and 0.5%, respectively. These findings represent a quick and easy method for choosing highly active water for the biomass plastic biodegradation test.

## Discussion

This study demonstrated the rapid erosion of PBSA in freshwater and brackish water RAS harboring Nile tilapia within 24 h. This erosion was confirmed as biodegradation in the holes, weight loss, SEM observations, DAPI staining, and real-time BOD test. BOD began to increase on days 1 and 2, coinciding with the erosion on the PBSA surface caused by microorganism activities. In addition, multiwell plate tests are useful for finding the optimum water condition for the biodegradation test within 48 h. Implementing a rapid biodegradation test using RAS water may prove possible in future studies as a rapid and straightforward method for selecting highly active water for biomass plastic biodegradation tests.

The biodegradable testing method usually requires a long period to observe the material biodegradation. Bioplastics do not easily degrade in nature; achieving their biodegradation, especially in water, is challenging (Mastrolia et al. 2022). PBSA biodegradability has been reported under various conditions and environments, including seawater, compost, garden soil, and culture medium. These findings summarized and compared to this study (Table 2), highlighted that RAS is an effective rapid tool for conducting biodegradation tests. In this study, microorganisms showed the ability to degrade 1 g/L PBSA by 11.6% in the 7-day trial (Fig. 5) in the RAS environment at pH 7.7, DO 7–8 mg/L, and water temperature of 25–26 °C (Table 1). The biodegradation test in the multiwell plate showed changes on the PBSA surface immersed in tilapia RAS water within 48 h, as described in the “Quick Biodegradation Test in a Multiwell Plate Using Water from Various RAS Tanks” section. According to the results obtained using PBSA, the rapidness of RAS biodegradation was due to several environmental factors such as pH, temperature, microbial community, moisture content, and salinity. The advantage of RAS water is the higher concentration of nitrogen compounds and the distribution of microorganisms compared to natural water. Furthermore, the limitations of the carbon source in RAS water result in microorganisms using PBAS as their exclusive carbon source. Several authors reported that microorganism activities are significantly hindered at pH < 5 compared to alkaline conditions (Hussein et al. 2015; Tamnou et al. 2021). Previous research conducted at 15–28 °C suggested that temperature plays an important role as an environmental factor influencing biodegradation rates (Pischedda et al. 2019; Tamnou et al. 2021). Despite the consistent apparent activation energy of the biodegradation reaction, indicating persistency, there is an impact on the metabolic activities of the associated mesophilic microbial communities. In addition, results from multiwell plates revealed that degradation proceeds faster in brackish water environments, suggesting that it may be correlated with bacterial activity. However, the study was conducted for 48 h in a multiwell plate for the rapid biodegradation test, and a long-term evaluation is still required to confirm the effects of different salinities on PBSA biodegradability in RAS. The evaluation of marine biodegradability under varying salinity conditions is essential, as microbial communities and degradation rates are influenced by salinity. In this study, initiation of biodegradation was assessed in RAS water. To enhance the ecological relevance of the findings, future studies should incorporate long-term biodegradation assessments across different salinity conditions, including seawater, brackish water, and freshwater, to better understand the influence of salinity on microbial degradation activity.

**Table 2:** Biodegradation of PBSA under different conditions

Shape of the PBSA	Environments	PBSA-degrading microorganisms	Conditions	Biodegradability method	Initial weight/size	Biodegradability (%)	Biodegradation period	Reference
Stick	RAS water	*P. pseudoalcaligenes*, *P. stutzeri*	pH 7.6, 25 °C ± 1 °C	BOD biodegradability (respirometry activity sensor system, incubated with stirrer)	1 and 10 g/L	11.6% and 5.7%	7 and 14 days	This study
Film	RAS water	*P. pseudoalcaligenes*, *P. stutzeri*	pH 7.8 ± 0.3, 25 °C ± 1 °C	Weight loss (immersed in RAS tank)	6.1 ± 0.3 mg	100%	6 days	This study
Stick	RAS water	—	pH 7.1, 26.9 °C ± 0.1 °C	Quick erosion test (multiwell plate test)	—	Small/large holes on the surface	24–48 h	This study
Film	Deep-sea floor, below a depth of 757 m below sea level	*Profundibacter*, *Hyphomonas*, and *Desulfobacula*	1.5–2.6 °C	Weight loss		< 22%	3 months	Omura et al. (2024)
Film	Seawater	—	25 °C	BOD biodegradability	6–7 mg		1 month	Omura et al. (2024)
Film	Culture medium	Lipases derived from bacteria	pH 7.0, 37 °C	Weight loss (incubated with shaking)	10–20 mg	100%	96 h	Huang et al. (2021)
Dog bone	Garden soil	—	pH 7, 30 °C ± 2 °C, moisture content of 55.6%	Mass loss	37.8 kg/mol	Initial period of biodegradation	After 16 weeks	Puchalski et al. (2018)
Dog bone	Compost	—	pH 7, 58 °C ± 2 °C, moisture content of 53.1%	Mass loss	37.8 kg/mol	Initial period of biodegradation	After 4 weeks	Puchalski et al. (2018)
Film	Culture medium	*Azospirillum brasilense*	pH 7.0 ± 0.5, 30 °C ± 2 °C, 50% ± 5% relative humidity	Weight loss	50 × 35 mm, 0.05 ± 0.02 mm thick	16%	30 days	Wu (2012)
Pellet	Activated sludge wastewater	*P. aeruginosa*, *B. cepacia* (culture cells isolated from activated sludge wastewater)	37 °C	Produced CO_2_ (modified Sturm test)	5 g/L	78%	40 days	Lee and Kim (2010)
Film	Soils and composts	*B. pumilus* (culture cells isolated from soils and composts)	pH 7, 30 °C (enriched culture liquid medium)	Weight loss (incubated with reciprocal shaking)	21 mg	100%	14 days	Hayase et al. (2004)

Analysis of the 16S rRNA gene sequence indicated that *Pseudomonas* (33.75%) played a significant role in the initial phase (24 h) of biodegradation in the RAS tank (see Fig. 6b). *Pseudomonas* is a diverse bacterial genus known for its ability to degrade various organic materials, including plastics. Some species play a key role in biodegradation by breaking down complex polymers (Wilkes and Aristilde 2017). In this study, two species from this genus were identified on the PBSA surface: *P. pseudoalcaligenes* (32.51%) and *P. stutzeri* (1.24%). *P. pseudoalcaligenes* has been reported to hydrolyze poly(butylene adipate-co-butylene terephthalate) (Wallace et al. 2017), highlighting its potential contribution to PBSA degradation. Meanwhile, *P. stutzeri* is known for its ability to fix nitrogen, perform denitrification under high DO conditions (Deng et al. 2014), and degrade polyethylene in slightly alkaline environments (pH ~ 7.5) at 26 °C (Tamnou et al. 2021). These properties aligned with the conditions in this study, where pH was 7.7 ± 0.3 and temperature was 25 °C ± 1 °C. In addition, *Pseudomonas* sp. has been shown to degrade thermoplastic polymers such as polypropylene in soil environments (Samat et al. 2023). These results suggested that *Pseudomonas* sp., which catalyzes ester bond cleavage and formation, plays a major role in the early stages of PBSA biodegradation. On day 35, *Methylibium* (33.74%) was the most abundant genus found on the PBSA surface. *Methylibium* belong to the class *Betaproteobacteria* and has been described as capable of hydrolyzing urea and reducing nitrate to nitrite (Nakatsu et al. 2006). These heterotrophic bacteria grow aerobically and are commonly found in water treatment systems and aquaculture environments. *Methylibium* is a lesser-known but functionally important bacterial genus that involved in the degradation of organic compounds. For example, *Methylibium petroleiphilum* PM1 is distinguished by its ability to completely metabolize the fuel oxygenate methyl tert-butyl ether (MTBE) and also degrades aromatic hydrocarbons present in petroleum products (Kane et al. 2007). In this study, the most probable reason for the erosion and weight loss of PBSA was the biodegradation by *Pseudomonas*, a bacterial process previously reported for degrading several kinds of biomass plastics (Lee et al. 2010; Wallace et al. 2017; Samat et al. 2023). In Fig. 2a, the weight of PBSA films decreased during 24 h (initial hydrolysis stage), as microorganisms secreted enzyme exposed on the film surface and enzymatic hydrolysis was initiated, resulting in the erosion of the PBSA film surface (see Fig. 3). The water continued to permeate the film, allowing enzymes located deeper within the film to access the water and initiate hydrolysis (Huang et al. 2021). As this process continued, the weight of the PBSA films decreased over time, indicating that 100% of the weight loss exhibited a complete degradation in the RAS tank at 144 h.

The BOD biodegradation test conducted using a respirometry activity sensor system VELP® in RAS water containing 1 and 10 g/L PBSA confirmed the degradation of microorganisms to CO_2_ within 1 to 5 days. Microorganisms utilized O_2_ for their metabolic activity to oxidize PBSA, a biodegradable organic material in RAS water, into CO_2_ and water during the exposure period (Takekoshi et al. 2022). By comparing the BOD biodegradation of PBSA at concentrations of 1 and 10 g/L, biodegradation in the 10 g/L group began earlier, on days 1 to 2, compared to the 1 g/L group, which started between days 2 and 5 of the test. The BOD concentration in the 10 g/L group was higher than that of the 1 g/L group by the end of the test, suggesting that 10 g/L is suitable for rapidly observing biodegradation. However, the degree of biodegradation in the 10 g/L group was lower than that in the 1 g/L group. Equations (1) and (2) can explain the low degree of biodegradation of the 10 g/L group due to the influence of the initial mass of PBSA on the degradation behavior. Specifically, the 10 g/L group reached the maximum BOD level (999 mg/L) in the device on days 14 and 21, with a biodegradation degree of ~ 6%. As shown in Table 1, DO levels in BOD bottles dropped to 3.45 and 0.75 mg/L, respectively, limiting the biodegradation activity by bacteria which leads to the underestimation of the true biodegradability of samples. The degree of biodegradation obtained in this study was lower than the OECD 301F standard, which classifies a substance as “readily biodegradable” if its biodegradability rate reaches at least 60% within 10 days of the 28-day test, mainly due to the high amount of PBSA substance used to evaluate BOD in this study. In a real RAS environment, where DO levels are not limited, the degree of biodegradation is likely to be higher than those observed under the conditions of this study.

The water in RAS does not fully represent a natural water environment but shares some similarities. As a controlled, closed RAS differs from natural water bodies in terms of oxygen levels, nitrogen compounds, and microbial communities. However, a key finding of this study is the presence of biodegrading bacteria in RAS, particularly those associated with Nile tilapia. This highlighted the potential role of RAS microbial communities in biodegradation processes of biomass plastics. Additionally, RAS can simulate certain aspects of nutrient-rich or eutrophic environments, such as coastal waters, estuaries, or aquaculture ponds, where organic matter and microbial activity are relatively high.

## Conclusion

The closed RAS was successfully applied as a rapid method for the biodegradation test. Real-time BOD test and SEM observations showed that bacteria in the RAS tank started degrading PBSA at 24 h. 16S rRNA gene sequencing identified *Pseudomonas* as the early decomposer in the biodegradation process within 24 h. The detection of bioplastic-degrading bacteria could be applied to solve the plastic pollution from the aquaculture industry, reducing plastic waste in the ocean. RAS is a highly effective tool for rapid biodegradation testing and holds significant potential for advancing knowledge and innovation at the intersection of materials science and biology in the future.

## Data Availability

No datasets were generated or analysed during the current study.
